# Privacy vs Usability: A Qualitative Exploration of Patients' Experiences With Secure Internet Communication With Their General Practitioner

**DOI:** 10.2196/jmir.7.2.e15

**Published:** 2005-05-31

**Authors:** Aksel Tjora, Trung Tran, Arild Faxvaag

**Affiliations:** ^3^Department of Neuromedical SciencesNorwegian University of Science and TechnologyTrondheimNorway; ^2^Department of Sociology and Political ScienceNorwegian University of Science and TechnologyTrondheimNorway; ^1^Norwegian Research Centre for Electronic Patient Records (NSEP)Norwegian University of Science and Technology (NTNU)TrondheimNorway

**Keywords:** Internet, patient-physician communication, electronic mail, qualitative research

## Abstract

**Background:**

Direct electronic communication between patients and physicians has the potential to empower patients and improve health care services. Communication by regular email is, however, considered a security threat in many countries and is not recommended. Systems which offer secure communication have now emerged. Unlike regular email, secure systems require that users authenticate themselves. However, the authentication steps per se may become barriers that reduce use.

**Objectives:**

The objective was to study the experiences of patients who were using a secure electronic communication system. The focus of the study was the users' privacy versus the usability of the system.

**Methods:**

Qualitative interviews were conducted with 15 patients who used a secure communication system (MedAxess) to exchange personal health information with their primary care physician.

**Results:**

Six main themes were identified from the interviews: (1) supporting simple questions, (2) security issues, (3) aspects of written communication, (4) trust in the physician, (5) simplicity of MedAxess, and (6) trouble using the system. By using the system, about half of the patients (8/15) experienced easier access to their physician, with whom they tended to solve minor health problems and elaborate on more complex illness experiences. Two thirds of the respondents (10/15) found that their physician quickly responded to their MedAxess requests. As a result of the security barriers, the users felt that the system was secure. However, due to the same barriers, the patients considered the log-in procedure cumbersome, which had considerable negative impact on the actual use of the system.

**Conclusions:**

Despite a perceived need for secure electronic patient-physician communication systems, security barriers may diminish their overall usefulness. A dual approach is necessary to improve this situation: patients need to be better informed about security issues, and, at the same time, their experiences of using secure systems must be studied and used to improve user interfaces.

## Introduction

It has been claimed that advances in information technology and computer literacy among the public have the potential to empower patients and transform health care [1]. The emergence of Internet and electronic communication links between physicians and patients is believed to have many potential benefits. Health portals, physician Web pages, and email channels for exchange of personal medical information allow for more complete and thoughtful health communication. This may, in turn, foster a new “breed” of health care consumers who slowly redefine the physician-patient relationship. Spielberg has suggested that use of email may enhance the level of intimacy shared between physician and patient, making their respective private spheres more accessible. For instance, patients who are reluctant to raise sensitive topics face-to-face or who seek a quick opinion between office visits may find electronic communication inviting [2]. Web-based programs may also provide chronic disease management support [3]. Moreover, many researchers have proposed that email has improved both access to and continuity of care [4] and has increased patients' involvement in their own care [5].

### Ambiguous Evidence on Electronic Patient-Physician Communication

Health care providers who generally experience a high demand for their services fear that their workload may reach an unsustainable level if they open a new communication channel [6,7], and they have also been worried about reimbursement issues [5]. Physicians communicate by email with only a very small proportion of their patients [8], but the selection criteria remain unclear [4]. Also, very few patients with email access actually use it to communicate with their general practitioner (GP) [9], and there is no unambiguous evidence to indicate for which purposes this communication is used. Yet, email has been used to communicate causes of symptoms, diagnostic test results, therapeutic interventions, and to obtain second opinions as well as general information on a specific disorder, treatment, or medication without reference to a specific patient [10]. According to Sittig, email messages from patients to providers include various requests for both information and action [11]. There is a possibility that some of these communications may have replaced a number of office visits [12]. However, the major problems with patient-physician communication via the Internet are the issues of trust, privacy, and legal concerns [13], even though it has been found that patients have been only mildly concerned with these issues [14].

### The Issue of Trust in Electronic Patient-Physician Communication

With the acknowledgement of the potential benefits of electronic communication, it has become an important aim for health care providers and government authorities to establish services that offer secure channels for health communication [15]. In order to be regarded as secure, a communication system must have mechanisms for message protection during transfer and storage. Further, it is mandatory that the users explicitly prove their identity (authentication). In electronic communication, those who participate cannot rely on the recognition of voices and faces to establish trust. It does not suffice to simply log on to one's household computer and start the email application. In practice, the user must go through a set of actions that establish a system user identity and link that identity to the actual identity of the user. The creation of a system user identity usually requires that the users physically identify themselves in front of a person who is authorized to register new users into the system. “The credentials” are a password, smartcard, or software token that is chosen or generated that the user will need in order to gain access to the system. The credentials must be transferred to a user before he or she can apply them to verify his or her identity with the system. A communication session can then be initiated.

### Secure Web-Based Communication Is Underused but Well Received

Communication systems that possess the above-mentioned security properties are gradually becoming available. Since they are quite new, little research that addresses their use by patients and providers is available. An electronic Internet link called the Patient Gateway has been identified as one system that offers a safe solution [8], and it has been well received by primary care clinicians [16]. In their study of a Web message service between GPs and patients, Liederman and Morefield reported favorable experiences of both care providers and patients [17]. A recent Norwegian study has reported on another secure Web-based solution called PasientLink [12]. In that study, however, only 48% of the intervention group had used the modality, while the non-users reported that they had felt no need for a doctor during the study period and that they did not regard the system as appropriate for the actual request [12].

### The Need for Addressing Patient Experiences

If care is not taken during the design and testing of systems that are developed for secure information exchange, the procedures required for authentication may become barriers that reduce use and, hence, overall utility of the system, although some “challenge” might be acceptable [7] since most patients are willing to accept a certain barrier for security reasons. Faced with applications that have poorly designed interfaces or that otherwise appear unfamiliar, appropriate and effective use by patients may not be achieved. Our aim was to explore the experiences of patients using a Web-based patient-physician communication system, with MedAxess as an example of such a system. We asked how participants used MedAxess, for what purposes, and what the results of that use were. We wanted to focus on information security issues from the users' perspectives and on how users perceived MedAxess as opposed to ordinary email in the same context. We were also interested in how strict regulations limit the use of MedAxess.

### Computer and Internet Availability and Use in Norway

Norway is well off into the information age. In the second quarter of 2004, as many as 60% of Norwegians had an Internet-connected personal computer (PC) available at home. Half of these people also had a broadband connection. A total of 79% of respondents had used a PC during the last three months [18]. On an average day in 2003, 42% of Norwegians were connected to the Internet for an average of 72 minutes [19]. In 2002, 45% of the Norwegian adult population reported that they might like to contact their family doctor over the Internet [20]. With regard to computer availability, skills, and a willingness to use electronic communication, the Norwegian population is similar to that of other industrialized countries [21].

### Patients and GPs in Norway

Most Norwegian GPs work in privately owned group practices. Primary medical care in Norway is organized through a patient list system that entitles every Norwegian citizen to be permanently listed with a local GP. The financial reimbursement is a mixture of a per capita annual fee from the National Health Services and a fee for service for individual consultations. Norwegian GPs have between 1100 and 2500 patients on their list, with an average of about 1280 patients [22].

## Methods

### The MedAxess System

**Figure 1:**
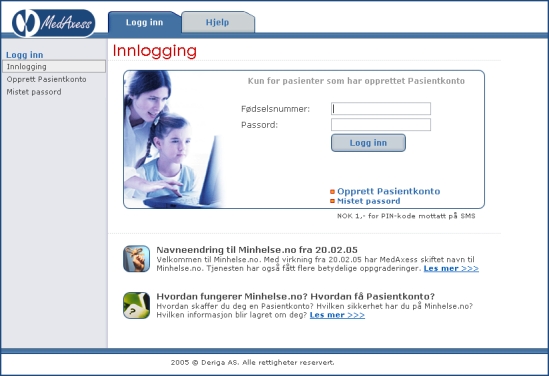
MedAxess log-in screen

**Figure 2:**
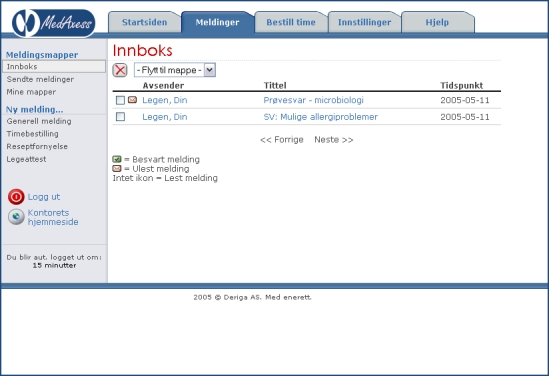
MedAxess inbox screen

**Figure 3:**
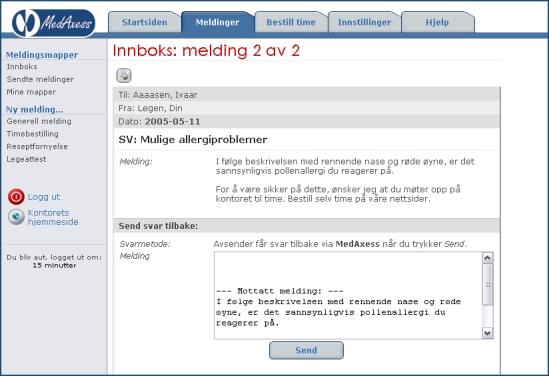
MedAxess message screen

MedAxess is a software system for secure exchange of information between a health care provider and a patient. It was developed in Norway by Deriga and has been piloted in primary care since 2002. The system has been approved by the Norwegian Data Inspectorate. In order to become a user, the patient must be registered as a list patient at the GP's office. Further, to be registered as a MedAxess user, the patient must choose a password from the GP's office. In addition to a PC connected to the Internet, the patient must also have a cell phone. Access to the system requires the user to open a Web browser and log on to the MedAxess “client” from the home page of the physician's office. After submitting the first password and passing the first log-on, in the second page, the user must request the system to generate a second, instant password to be sent to his or her cell phone as an SMS message. Once this procedure has been completed successfully, the user can transmit and receive messages with the GP. The MedAxess log-in, inbox, and message screens can be seen in Figures 1-3.

### Study Design

This study was based on interviews of MedAxess users who were listed as patients at one GP office in an urban setting in Trondheim, Norway. When the study took place in October 2002, 70 listed patients were registered as MedAxess users; however, four months after it had been implemented, only 35 of them had actually used the system. Based on availability when the GP's secretary called, 15 patients who expressed their willingness to participate were selected and recruited from among these 35 users. When the interviews took place during the spring of 2004, the 15 selected patients would have used MedAxess for about 12 to 18 months.

All respondents were interviewed using a semi-structured design. The interviews took place at the Norwegian Research Centre for Electronic Patient Records (NSEP) (13/15) or at the patient's workplace (2/15). They lasted from 15 to 40 minutes, with a typical duration of 30 minutes.

All interviews were tape recorded, fully transcribed, and, initially, manually analyzed by the second author. The first author then analyzed the interview transcripts independently using the NUD-IST qualitative analysis software and applying a grounded theory approach by which empirical data are thematically categorized by induction [23]. To increase validity, results of the two independent empirical-analytical approaches were compared by all authors. In the first analysis, five main themes were directly identified from the transcripts: (1) patients' attitudes toward security, (2) aspects concerned with exchanging information with MedAxess, (3) easier access to the doctor, (4) unwanted incidents, and (5) perception of ease of use. In the second analysis, 38 themes (or categories) were initially identified and then sorted into six main themes: (1) supporting simple questions, (2) security, (3) aspects of written communication, (4) trust in the GP, (5) simplicity of MedAxess, and (6) trouble with MedAxess. The themes from the first and second analysis, although differently grouped, extract the same issues from the empirical material. This was taken as a confirmation of the grounding of the analysis in the data.

This paper reports on all themes, using the six-theme grouping from the second analysis. Extracts from interviews are indexed pX/Y, where X is the informant (patient) number and Y is the text segment number within that interview. These numbers are used for internal tracking purposes in order to be able to refer interview extracts back to the context of raw data, for example, in relation to later discussions of the paper.

## Results

Six different but related findings could be extracted from the interviews: (1) patients experienced easier access to their GP by using MedAxess, (2) patients tended to solve minor problems with their GP, (3) patients elaborated on larger issues with their GP, (4) patients did not worry too much about information confidentiality, (5) patients experienced the log in as awkward and a barrier to use, and (6) some patients preferred plain email instead of MedAxess.

### Patients Experienced Easier Access to Their GP

An overall reason to use MedAxess is that patients gain easy access to their GP. Through MedAxess patients may get in touch with their GP without having to wait on the phone or arrange a consultation. In particular, for users with significant travel distance to their GP's office, the possibility to communicate in this manner is “an extra bonus.”

p14/8 I live in another part of town than the GP's office. And, in addition, I would rather not leave work to sit and wait in his office, like it used to be, to have a prescription or an appointment or something else. Now I can do all these tasks on the net, and I have a response the same day. I think it works great.

Patients mostly have limited direct phone access to their doctor since most physicians spend their time in consultations and have secretaries to answer and screen the majority of incoming calls. Some practices have organized certain telephone time slots during the day when GPs will answer questions directly from patients. However, since a vast number of patients will try to contact the doctor during that short time slot, such telephone hours often result in long waiting times or no response whatsoever.

p5/36 Then it is very hard to get the GP on the phone. The hours with telephone access is very limited, so I have tried to use it, but you have to plan carefully. It is one hour, maybe two or three times per week, and then you must be sure to call during that hour. And the line is not necessarily available. Now you just have to write and then he will answer when it suits him.... So it [MedAxess] is very convenient.

Most of the MedAxess users (10/15) reported that their GP responded surprisingly quickly to their questions and requests. This means that the patient may contact the doctor asynchronously, without having to wait on the phone, and still get an answer to short questions within minutes. As a consequence, a number of the users (8/15) felt that the GP was more easily accessible through MedAxess than he or she would be otherwise. However, 2 of the 15 respondents reported that they did not get any response from their GP on MedAxess, without being able to explain why.

### Patients Solved Minor Health Problems by Using MedAxess

Although there was a tendency for patients to prefer using MedAxess for minor problems, some patients reported that it was convenient to use it for specific problems that they felt were too complicated to explain on the phone or that required some interaction back and forth between patient and doctor. The time constraints in regular office and telephone consultations rarely allow for in-depth discussions, and they tend to limit the opportunity for the patients to reflect on the GP's suggestions.

p12/8 You are supposed to say everything on as little time as possible and be very precise right then. But via that system [MedAxess], you could ask for advice and perhaps a bit of background and spend some more time when you want to ask a question, and to present what is important to get through.

MedAxess may help users to reflect on suggestions given by the GP since it provides asynchronous communication. Also, the written communication may make a significant difference in establishing a dialogue between the patient and physician.

p14/32 It is much easier than going [to the GP] to sit down to wait for an appointment. So I think I feel that the contact with the doctor someway has been better because you have more dialogue, so to speak, on the small matters. Then you are a little more confident about the larger matters.

Even though any text-based communication like that of MedAxess is qualitatively different from a face-to-face or telephone interaction, it is the asynchronous nature of the text-based communication that gives users the chance to take the care and time to present a more comprehensive request or question to the doctor.

### Patients Used MedAxess to Elaborate on Complex Health Problems

One of the most interesting aspects with an asynchronous communication system like MedAxess is the potential not only for short questions but also for longer descriptions of health problems. Patients reported that, with MedAxess, they were able to elaborate on illness experiences and also make their own suggestions without feeling that they used too much of the physician's time.

p12/8 When you call the doctor in the telephone time you know you have limited time. One is supposed to speak only briefly and be very concise there and then. But through the MedAxess system, you could ask for advice and perhaps a bit more background and take your time to ask questions and get through with important matters and so on.... I try to include everything that is relevant. The other day I wrote that I had such and such symptoms and I needed to include some background history, that I have been examined for this that year. Then I try to give a complete picture of my health, then and now, enough for the physician to sort of grasp the continuity.

Many patients reported that the written communication gave them the opportunity to think carefully through their message, for example, their illness history, as described by the informant above. Using text to communicate provided a less stressful situation, allowing patients to produce a full illness narrative. Some patients felt that there was always too little time to talk with the GP during office consultations.

p14/36 You have a feeling that things move fast here [at the GP's office]...and that, even if the doctor does not think that way, you think since you got a consultation in between other patients...he is in a hurry. And it ends up in such a way...that you think afterwards, “Oh, I should have said that. I forgot!” But when I use the Net, I have time to think through how to formulate and describe things.

Other users reported that using MedAxess for complex medical problems was useless since text-based communication is not a rich enough medium to reach an understanding between doctor and patient. These users meant that electronic communication was too impersonal for substituting the face-to-face consultation. However, users would, at the same time, argue that patients who knew the doctor well would be able to use electronic communication with greater success.

Another aspect with the written communication that was reported by the MedAxess users was the chance to suggest a medical analysis themselves. Patients with chronic illness, especially, are often well educated and may have the ability to suggest some therapeutic interventions to the doctor.

p15/28 I have so-called autoimmunity and have had to learn to refer to my own illness or health. So, because of that, I guess I have internalized a terminology and an attitude towards not going to the doctor just to tell him that I have some pains. I try to analyze, myself, so therefore I am a bit specific in my descriptions.

A question related to privacy issues is how users think about sensitive issues being communicated via the MedAxess system. This is slightly ambivalent: many patients perceived MedAxess as useful for simple messages regarding appointments, prescriptions, and so on; however, other patients utilized the tool to discuss sensitive matters.

p8/7 It becomes more private. You know, you want to discuss in private with a doctor, and you can write, and you feel that it is more directly from you to him.... You, in a way, dare to write a bit more on such [a system].

p7/26 I do not think it is a problem to write about things that I am worried about. It has not been a problem at all.... Even in some cases, I would think that if there is something that is really difficult to talk about, perhaps it would be easier to write about it.

The potential of the MedAxess system to let patients elaborate on illness experiences, even those where a high level of privacy is expected, might be an important finding in a time when complex chronic illnesses that might need to be thoroughly discussed between patient and provider represent a large portion of health care provision.

With the MedAxess system, it was also found that the GPs had more time to respond to difficult questions (as long as time was available to spend). GPs were in control of the response time and, therefore, in the long run, were also in control of the patients' expectations of response time. The doctor was therefore able to either think thoroughly through alternatives or use additional resources to make a decision.

p5/20 I had a question regarding some natural medicine that I had started using without consulting the doctor. And then I was told that I should not use it and it was in a way a bit acute [urgent]. [The system] was very convenient because I explained the situation and received a very thorough answer that I would not get if I asked him in a consultation. He had forwarded the question to a research institute for natural medicine and received a response that he forwarded to me. So I received information from this source directly, with an answer, and it went quite fast.

In summary, the fact that MedAxess let patients communicate with their GP through text provided an opportunity for patients to present illness experiences in a more relaxed way, with possibilities to elaborate on sensitive topics and include historical and contextual information, as well as patient hypotheses. The GP would also have the chance to check with external expert resources before providing an answer to the patient. Some users would argue that a personal relationship between the doctor and patient should have been established before an extensive use of electronic communication substituted telephone and face-to-face communication.

### Patients Were Not Too Concerned About Information Confidentiality

One main achievement of MedAxess is that it satisfies the strict health information security regulations in Norway and other European states, as mentioned previously. The interviews have documented, however, that patients were, in general, not especially worried about confidentiality. When the patients personally assessed the information security of MedAxess, they often made a comparison with economic transactions on the Internet. Many MedAxess users had favorable experiences with several years of Internet use, and one patient compared MedAxess with the use of Internet banking services.

p3/80 We are used to transferring money over the Internet...in and out of Internet banking services. So, if you are afraid of being watched—I am not, but I understand that people might have problems with that—it seems paralyzing. That fear may be paralyzing for information transfer in general.... So, MedAxess is a good thing, to my opinion. And then you have to take chances [laughs].

One important aspect of the users' perceptions of security (ie, confidentiality) was that their immediate experience with the rigorous log-in procedure elicited the feeling of a high security level. The users expected that the only reason for the awkward procedures must be security issues, and that these issues were necessarily addressed by the procedures.

p13/9 I feel that it is safe because it is like this: I receive a new password every time, which they transmit to my mobile phone. So I hope that this means it is safe...that the passwords are stored in another system.

Most respondents were conscious about the security problems on the Internet and thought that information transactions could never be 100% safe. There are several reasons why users were relaxed about using MedAxess. First, they regarded personal health information as of limited interest to the potential hacker. Second, the users were extra cautious not to elaborate their most intimate details during communications via MedAxess.

p11/92-95 It has only been questions about when to start with [an] allergy medication and that kind of general matters. I do not care if people should learn about that.... When it is something serious, that is something you don't email.

p10/29 I would not like to discuss my health over the Internet; I never would have. That confidence in the net, I would never have. But that is not the point either. If you are really sick and need to talk to the doctor, then you should talk to the doctor and not sit there chatting on email, sort of. So I think it's fine.

However, some patients were uncertain about a potential misuse of information transmitted through MedAxess. Also, the fact that communication is logged and stored in a database made the situation quite different from that of, for instance, telephone conversations. If such written communication is stored for a very long time, it is difficult to foresee who will have access to the information in years to come.

p12/33 You have that feeling, when you push the send button, “Well, well”; you hope what has been said about it being absolutely secure is really true.

p12/75-76 But it is obvious that the incidences stay there, the history, and you may see which questions were asked one year ago. And the doctor has the same log. But I guess you have to trust that nobody else has access.... Since [communication] is stored...the thing about security and safety strikes me.... You are aware that it is not erased, you know.

Being sceptical of applying MedAxess for complicated or intimate health issues is not only related to the security concern. Some users reported that the limits of text-based electronic communication make MedAxess less useful for comprehensive discussions. They would rather elaborate on personal issues with the GP face-to-face, watching the doctor's verbal and physical response closely, than to have immediate feedback.

### Logging In to MedAxess Was Awkward Compared to Email

As reported by the users, the awkward log-in procedure was a main problem with MedAxess. Users had to submit a message from their computer, wait for a pin code to be sent as an SMS message to their mobile phone, and then submit that pin code on the computer to get access to the system. The trouble of “passing the security obstacles” seemed to limit the amount of frequent users of MedAxess.

p15/112 I had been to the doctor to take some tests. I wanted to have the results and had problems with accessing MedAxess.... So I called the GP's office and then got a combination of numbers.... I tried one more time, without success. I asked again at the office and they told me to call them or those [technically] responsible. I can't remember their name. It became too awkward. Since then, I haven't thought too much about it.

To comply with the Data Protection Act, MedAxess is based on a Web interface instead of an ordinary email account. This means that users cannot just check responses during the same operation as when they check their other email. Thus, they have to log on to MedAxess separately to check for answers from the GP's office. In particular, users who read email as part of their regular work could have saved a lot of time if they were able to access their communication with the GP by using ordinary email.

p13/58 When you use such Net-based systems that have nothing to do with your email account, you have to access it separately. And I read so much email for the rest of the day or do so many other things, that to log on to check if I have had a response today—I don't bother. Then, it is much better to use an email account that I use on a daily basis.

These problems have led many patients to use ordinary email instead of MedAxess. The GP offices in this project had communicated non-sensitive issues by email with some of their patients for many years before MedAxess was introduced. Beforehand, some patients had therefore been used to email communication with their care provider in a way they had found useful.

p14/24 If it is those quick things that I need, I just send an email to the reception. If I need a prescription...I send an ordinary email because they have some sort of prescription ordering where it is just [necessary] to contact the office desk. So, I use MedAxess more directly when it concerns my disease.

The preferred use of email rather than MedAxess may be understood as a reaction to the awkward security procedures related to the system. It may also be a result of a patient's well-established routine of using email with the doctor.

p13/30 It was the last week or the one before that. It was a... patient record note that was written by a psychologist that was totally far out, the way I saw it, that [my doctor] got a copy of...and I sent her an email where I asked her to look at the note and give some feedback if she agreed. She would look at the matter. And she replied after two days. That was rather quick, I think.

Moreover, a reasonable interpretation of patients' use of email instead of MedAxess is their relatively relaxed attitude toward confidentiality problems with email in general. Although the patients acknowledge that these security issues have been solved within MedAxess, they often make their own judgment as to whether the content of their communication is suitable for email even when they have access to MedAxess. It is quite interesting that the users assessed on their own behalf the privacy content of the information they transmitted. Thus, it challenges the role of the Information Security Act as well as the security functions of systems like MedAxess. To avoid too much hassle with logging in, some patients selectively preferred to use email for “small practical matters.”

p12/17 I actually sent an email once more. I took that road again, the question related to a test result on my daughter that I was supposed to report. But then I didn't try [MedAxess]. I used ordinary email because it was much easier, since I knew there had been some trouble getting access the last time. But if I had the chance, I would rather use [MedAxess]. Because I regard it as a more secure and direct access to the doctor than the GP office's email address.

The patients are not especially concerned about security issues on the receiver side (ie, at the GP's office), and they regard the GP as reliable when it comes to who reads the office email.

p13/15 Those simple things like ordering Paracet or asthma medicine, I could of course use [MedAxess] for that. But it hasn't turned out that way. I have sent [my doctor] an email.... It...seemed easier for me, at least. And I got a reply at once. Certainly, I hope they have a safe email system, that it is encrypted so that it is not possible to hack the system. So I had to rely on that. It is a [technologically advanced] GP office...to my experience, so I hope that's in place. Anyway, I got a response, very quick.

The main reason for using email is the awkward log-in procedure of MedAxess. In addition, regular email is convenient for the respondents who use email on a daily basis at work.

p2/18-20 I have sent...an ordinary email, yes, because I sit in front of the computer all day at work, and at first I discovered that I could send an email to request an appointment.

It is interesting to note that email seems to have established itself as an ideal standard for user-friendly computerized communication.

p12/72 And if it turns out to work as fast as email, I will use it for matters for which I could have used email.

Our empirical data have shown that 5 of the 15 users preferred email for communication with their GP. In doing so, they avoided some of the log-in hassle and also made it possible to integrate their communication with the GP with other email-based work activities. Of special interest here is the finding that users made a self-assessment of their privacy need to distinguish between different kinds of communication media use. Even though they regarded email as less secure than MedAxess, they chose to use email because of its ease. At the same time, however, they made sure that the information they submitted via email was of a less private nature.

## Discussion

This study was limited to a qualitative approach with a focus on the various experiences of patients using MedAxess. Thus, we have taken an explorative approach to identify issues concerning how users perceive information privacy matters and how they act accordingly.

### Reasons for Using MedAxess

Patients used MedAxess for “small matters,” which they did not regard as particularly sensitive. They avoided the most intimate details and therefore reduced the relevance of confidentiality worries. Examples of reasons for using MedAxess included the following: to ask for recent test results, to request documentation such as renewed prescriptions and certificates, and to give feedback on results of medications taken at home.

In addition, patients found MedAxess useful for elaborating on larger issues, for example, concerning their experiences of changes in a chronic illness situation. The fact that MedAxess provided an asynchronous text-based medium gave patients an opportunity to present their story without feeling stressed because they were using the GP's time.

Users regarded MedAxess as making access to the GP easier. They did not have to travel to the GP's office or queue up in a long phone line. They felt that they did not have to disturb the GP with small questions. The response time from the GP was reported to be fast, sometimes surprisingly fast.

### Reasons for Not Using MedAxess

Several users (6/15) regarded MedAxess as not quite user friendly and therefore used MedAxess quite infrequently. This resulted in difficulties recalling the cumbersome log-in procedure. Some of these users ended up using ordinary email for communication with their GP, thus avoiding some of the log-in hassle and making it possible to integrate electronic communication with their GP with other email-based work activities. Users who relied on regular email regarded it as safe enough for the kind of information they communicated to their GP.

### Security Issues

About half of the users (7/15) in this study perceived MedAxess as secure because of the awkward password system (“Why else would one have it this way?”), because it was planned with information security in mind (and supposed to be more secure than email), and because it had passed the strict regulations of the Information Security Act. Users had already used Web-based banking services without many second thoughts and therefore knew that Web services might be safe. Supporting the findings of Hassol et al [14], patients in this study were only mildly concerned with information security issues.

### Privacy Issues

As expected, we identified that users were interested in applying MedAxess for small, practical issues, and that they found their GP to be easily available through this system. However, the perception of privacy issues among users was more surprising. To avoid the log-in hassle of MedAxess, they preferred to use ordinary email, avoiding security problems through some self-assessed adjustment of the information they transmitted. It seemed to bother patients less thatit is illegal for doctors in Norway to give medical advice to their patients via ordinary email. According to Norwegian regulations, the doctor is responsible for responding to such messages if it is expected that the problem described needs medical attention or treatment. In that situation, the GP would have to ask the patient to make an appointment or use a secure system, such as MedAxess, if the Internet is the obvious avenue to discuss the problem at hand. Or, the GP could simply call the patient on the telephone or ask the patient to call.

### Conclusions

With email as an ideal, the challenge for secure Web-based communication systems is to establish log-in procedures that users will find easy, effective, and feel familiar with. As mentioned by Masys et al, safety comes with a price in usability, which might even be acceptable [7]. Moreover, as we have demonstrated in support of the findings of Masys et al [7], the technical challenge of using the system contributes to the perception of safety.

On the other hand, our results clearly show that the usability of the log-in procedure has an impact on patients' actual use of the system. Only half of those patients who registered as users of MedAxess actually started using the system. Our results are based on responses from these patients; therefore, patients in our convenience sample might have more positive attitudes towards MedAxess than the average patient. Taken together, these results emphasize the need to address usability issues when developing and testing such systems. Perhaps there might be a need to educate users more on security issues before it is possible to widely implement systems that cannot necessarily be as easy to use as regular email.
